# Pediatric trochleitis associated with paranasal sinusitis: a case report

**DOI:** 10.1186/s12886-019-1030-4

**Published:** 2019-01-14

**Authors:** Hyunkyu Hong, In Jeong Lyu

**Affiliations:** Department of Ophthalmology, Nowon Eulji Medical Center, Eulji University School of Medicine, #68 Hangeulbiseok-ro, Nowon-gu, Seoul, Republic of Korea 01830

**Keywords:** Trochleitis, Trochlear pain, Sinusitis, Children

## Abstract

**Background:**

Trochleitis is trochlear pain with evidence of inflammation in the trochlear area on radiologic examination. The etiology of trochleitis is mostly idiopathic. Secondary trochleitis is rare, and trochleitis associated with paranasal sinusitis in children has not yet been reported.

**Case presentation:**

An 8-year-old boy presented with left periorbital pain for a week. His visual acuity and eye movement were normal. There was point tenderness on palpitation over the left trochlear region without swelling or redness. Orbital magnetic resonance imaging showed focal enhancement on the left trochlea and paranasal sinusitis on the left side. The patient’s symptoms and signs were completely resolved after empirical treatment for sinusitis. There was no need to inject a local steroid.

**Conclusion:**

Although rare, sinusitis should be considered when diagnosing and treating trochleitis in children with periorbital pain.

## Background

Trochleitis is the local inflammation of the superior oblique tendon trochlea. The diagnosis of trochleitis is based on clinical findings and radiologic images. It is characterized by superomedial orbital pain and point tenderness on palpitation over the trochlear region.

Trochleitis is mostly idiopathic. Secondary trochleitis is rare and is associated with systemic autoimmune disease or lymphoma [1, 2]. Herein, we report the first case of a pediatric patient with trochleitis associated with sinusitis.

## Case presentation

**Fig. 1:**
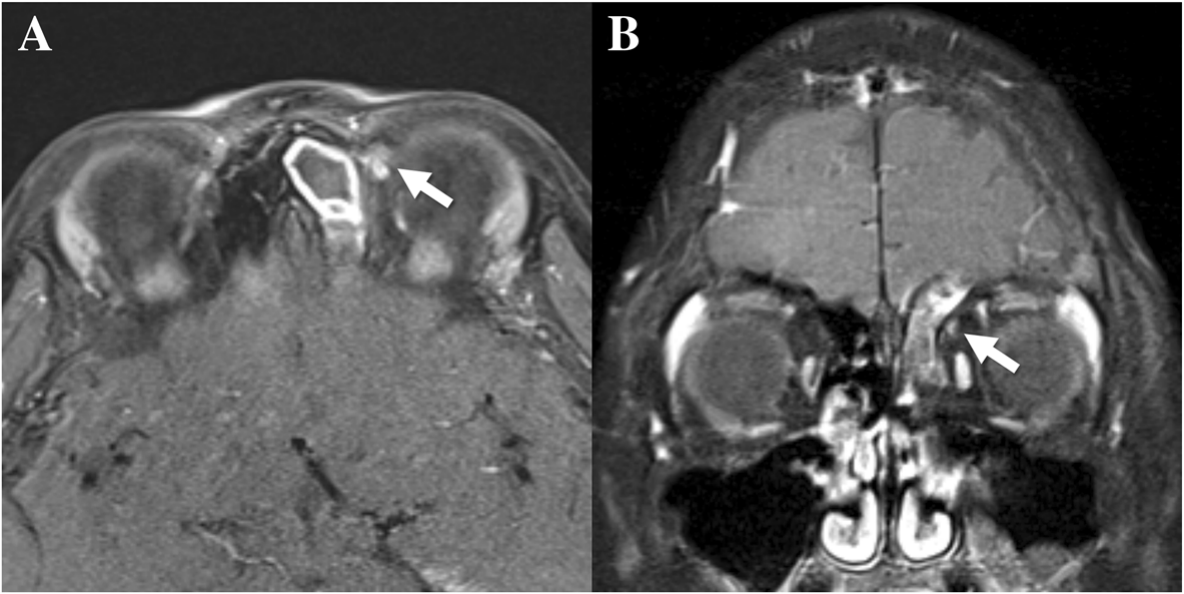
Gadolinium-enhanced T1 MRI demonstrating enhancement on the left trochlea (arrows). Frontal, ethmoidal, and maxillary sinusitis is also observed. **a** axial image, **b** coronal image

An 8-year-old boy presented with left periorbital pain for a week. His parents reported that he had mild headache intermittently when he contracted upper respiratory infections but was otherwise healthy. His uncorrected visual acuity was 20/20 in both eyes with mild hyperopia. Ductions and versions were normal, although there were 10 prism diopters of intermittent exotropia as determined by prism and alternate cover testing. There was no pain on eye movement. However, the patient presented intense tenderness on palpitation over the left trochlear region without swelling or redness around the left periorbital area. Orbital magnetic resonance imaging (MRI) showed focal enhancement on the left trochlea (Fig. 1). Left frontal, ethmoidal, and maxillary sinusitis was also detected. The patient, however, had not been previously diagnosed with sinusitis. We referred the patient to an otorhinolaryngologist. On rhinoscopic examination, the patient presented mild rhinorrhea with posterior nasal drip, and the mucosa was swollen in the left middle meatus. He underwent treatment with oral empirical antibiotics (amoxicillin/clavulanate syrup for 9 days and then cefpodoxime syrup for 4 days), leukotriene receptor antagonist, and steroid nasal sprays to control the sinusitis and rhinitis. The symptoms and signs were completely resolved after a course of treatment without the need for local steroid injection in the trochlear area. There was no recurrence during the 8-month follow-up period.

## Discussion and conclusions

Trochlear pain is subclassified into primary trochlear headache, trochlear migraine, trochleitis, and inflammatory Brown syndrome [1]. Primary trochlear headache is a specific headache localized in the trochlear region without evidence of inflammation [3]. Trochlear migraine is diagnosed when exacerbation of the trochlear pain triggers a migraine attack [1]. Trochleitis is trochlear pain with evidence of inflammation in the trochlear area on radiologic examination [4]. Finally, inflammatory Brown syndrome is trochleitis accompanied by the limitation of the upward gaze of the affected eye in adduction [5]. Trochleitis is one of the differential diagnoses of headache and orbital pain. However, it is poorly recognized and often misdiagnosed as many clinicians are unfamiliar with this condition. Trochleitis is characterized by pain and tenderness in the involved trochlea, and this condition is often worsened by elevation. Swelling and inflammatory changes are demonstrated on radiologic evaluations such as computerized tomography (CT), MRI, and ultrasonography [4].

Most cases of trochleitis are idiopathic and usually occur unilaterally [6]. Continuous friction of the superior oblique tendon through a trochlea generates chronic microtrauma, which results in stenosing tenosynovitis [1]. Secondary trochleitis associated with rheumatologic and immunologic disorders, including systemic lupus erythematous and rheumatoid arthritis, can develop [1, 7]. Because the majority of cases involve non-infectious inflammation, oral non-steroidal anti-inflammatory drugs (NSAIDs), systemic corticosteroids, and local steroid injections show favorable outcomes [3–5]. To the best of our knowledge, trochleitis associated with paranasal sinusitis, however, has not been reported, despite the anatomical proximity between the paranasal sinus and trochlear nerve. Only one case of inflammatory Brown syndrome after frontal sinus surgery has been reported [8]. Trochleitis in children has never been reported. However, orbital infections including orbital cellulitis and orbital abscesses are well known and common complications of sinusitis in children [9, 10]. Incomplete paranasal sinus development and thinner bony barriers in this age are thought to be the main reason [11].

In our case, the child with paranasal sinusitis presented with focal isolated inflammation in the trochlea area. His symptoms resolved after 2 weeks of empirical treatments for sinusitis and rhinitis. There was no need for local steroid injection or oral corticosteroid treatment, which are considered as the treatment for general trochleitis. Ruling out paranasal sinusitis is recommended before initiating oral or local corticosteroid treatment because the immunosuppressive effects of steroids may worsen infections.

To the best of our knowledge, this is the first report of isolated trochleitis associated with paranasal sinusitis in a pediatric patient. Although rare, sinusitis should be considered when diagnosing and treating trochleitis in children with periorbital pain.

## Abbreviations

CT: computerized tomography
MRI: magnetic resonance imaging
NSAIDs: non-steroidal anti-inflammatory drugs
